# Chemerin's Role in Endometrial Dysfunction: Insights From Transcriptomic Analysis

**DOI:** 10.1111/jcmm.70417

**Published:** 2025-03-21

**Authors:** Ming Yu, Yichun Wang, Jinxuan Cai, Xinyue Dong, Hao Wang, Zichen Sun, Tianxia Xiao, Jie Chen, Mengxia Li, Chunhua Shan, Yang Dong, Jian V. Zhang

**Affiliations:** ^1^ Center for Energy Metabolism and Reproduction, Shenzhen Key Laboratory of Metabolic Health, Shenzhen Institutes of Advanced Technology Chinese Academy of Sciences Shenzhen China; ^2^ Department of Medical Oncology The Fourth Hospital of China Medical University Shenyang Liaoning China; ^3^ Shenzhen College of Advanced Technology University of Chinese Academy of Sciences Shenzhen China; ^4^ College of Life Science Northeast Forestry University Harbin China; ^5^ Department of Obstetrics and Gynaecology, School of Clinical Medicine, LSK Faculty of Medicine The University of Hong Kong Hong Kong China; ^6^ Faculty of Pharmaceutical Sciences Shenzhen University of Advanced Technology Shenzhen China; ^7^ Sino‐European Center of Biomedicine and Health Shenzhen China

**Keywords:** 12Z cells, adipokine, chemerin, endometrial receptivity, transcriptomic analysis

## Abstract

Endometrium, the lining of the uterus, changes dynamically in response to fluctuations in ovarian hormones. The proper endocrine environment regulates endometrial functions: menstruation and supporting pregnancy. Obesity is closely related to endometrial dysfunction, which seriously affects women's health and fertility, but the pathological mechanism is unknown. Chemerin is an adipokine involved in multiple biological events such as immunity and metabolism by acting on its functional receptors. This study aimed to characterise the effects of chemerin on human endometrial epithelial cells by RNA‐Seq. 12Z cells were utilised as the model because immunoblot results showed that they expressed endometrial markers, epithelial markers and functional receptors for chemerin. Principal component analysis (PCA) showed that chemerin treatment significantly altered the transcriptome. Differential Expression Analysis found 388 significant differentially expressed genes (DEG) in the chemerin treatment group compared with the controls. Gene Set Enrichment Analysis (GSEA) showed that chemerin inhibited lipid metabolism and induced the epithelial‐mesenchymal transition (EMT)‐like process and cellular senescence. More importantly, GSEA and immunoblots showed that chemerin restrained the STAT3 signalling pathway, which is required for endometrial receptivity establishment. Collectively, these findings provide new evidence that over‐produced chemerin underlying the endometrial dysfunctions in obesity.

## 1 Introduction

Endometrium, the mucosal lining of the uterus, plays a vital role in receiving the embryo and maintaining the subsequent pregnancy [1]. For the woman at reproductive age, every month, endometrium dynamically changes at the molecular and structural level in response to fluctuations in ovarian sex hormones (mainly oestrogen and progesterone) across the menstrual cycle. Before ovulation, the endometrium continues to grow and thicken under the domination of oestrogen, which is termed the “proliferative” phase (equal to the ovarian “follicular” phase). Following ovulation, progesterone secreted by the remaining corpus luteum antagonises oestrogen‐mediated proliferation and transforms the endometrium to acquire the ability to accept the embryo, and this stage is termed the “secretory” phase (equal to the ovarian “luteal” phase) [2]. Embryo implantation occurs at a limited period, usually in the middle of the “secretory” phase, also known as the “Window of implantation” (WOI), during which an embryo attaches to the endometrial epithelium followed by penetrating and infiltrating into the endometrial stroma to establish the pregnancy [3]. If there is no embryo, progesterone withdrawal triggers the shed of the functional layer of the endometrium (menstruation), and the retained basal layer repairs the endometrium and proceeds to the next cycle [4].

Endometrium is a multicellular tissue that contains epithelial cells (luminal and glandular epithelial cells), stromal cells, vascular endothelial cells and immune cells [5]. Luminal epithelial cells line the endometrium, which direct contact with the embryo during WOI [6]. Glandular epithelial cells are invaginations of the epithelial cells extending from the lumen into the stroma, forming tubular glands, which perform secretory function [7]. Stomal cells differentiate into decidual cells under the control of progesterone, a process called decidualization, which is essential for embryo implantation and placentation [8]. These endometrial and non‐endometrial cells communicate and cooperate to complete several key physiological events such as re‐epithelization, menstruation, decidualization and embryo implantation. As reported, endometrial factors account for two‐thirds of cases of implantation failure [9]. Dysfunctional endometrium also leads to multiple diseases such as abnormal uterine bleeding (AUB), Ashman syndrome, endometriosis, adenomyosis and even endometrial cancer. These diseases often seriously affect female fertility with limited treatment options [10].

Currently, worldwide increasing obesity is closely linked with a higher incidence of subfertility of both sexes and 13 different types of cancer including endometrium cancer [11, 12]. Obesity causes endocrine, metabolic and inflammatory alterations that may be disruptive to the reproductive system. Clinical data showed that overweight and obesity do not seem to affect embryo morphology, blastocyst formation and quality, but negatively affect the outcome of natural and assisted conception, pointing to an alteration in the uterine endometrium [13]. Meanwhile, transcriptomic analysis revealed that obesity affects the endometrial transcriptome in both humans and mice [14, 15, 16, 17]. Animal models have also found that diet‐induced obesity affects uterine repair, endometrial decidualization, embryo implantation and endometrial hyperplasia [18, 19, 20, 21]. However, the detailed molecular mechanism underpinning obesity‐impaired endometrial functions remains unclear.

Adipose tissue secretes a variety of bioactive peptides called adipokines, which regulate physiological and pathological processes by acting on multiple target organs throughout the body [22]. Chemerin, the product of *RARRES2* (retinoic acid receptor responder 2), is mainly released by adipocytes, and its systemic level is increased in obesity [23]. The secreted 143 amino‐acids protein (pre‐chemerin) has low biological activity, and the bioactivity is determined by protease cleavage at the C‐terminus. Until now, three receptors of chemerin have been identified: chemokine‐like receptor 1 (CMKLR1), G protein‐coupled receptor 1 (GPR1, also known as CMKLR2) and chemokine CC‐motif receptor‐like 2 (CCRL2). CMKLR1 and CMKLR2 are functional receptors for chemerin, whereas CCRL2 is a non‐signalling receptor in response to chemerin [24]. Chemerin and the cognate receptors play vital roles in reproductive health and diseases [25], however, the effects of adipokine chemerin on endometrial cells are incompletely elucidated. In this study, we endeavoured to comprehensively identify transcriptome level changes of human endometrial epithelial 12Z cells after chemerin treatment.

## 2 Results

### 2.1 Chemerin System Expression in Human Endometrium Across the Menstrual Cycle

**FIGURE 1:**
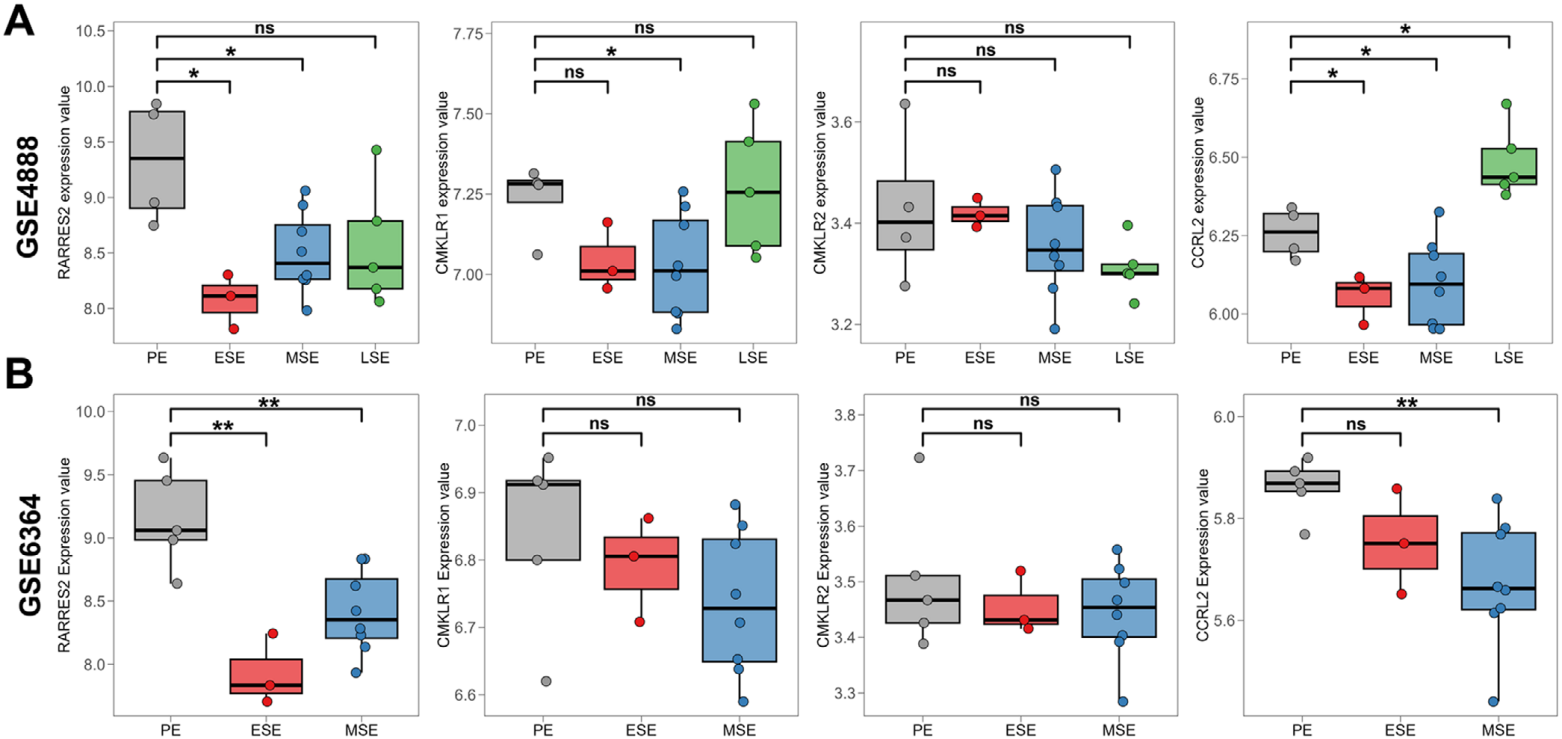
Gene expression of chemerin and the cognate receptors in healthy endometrium across the menstrual cycle. Gene expression levels of RARRES2, CMKLR1, CMKLR2 and CCRL2 in uterine endometrium at proliferative phase (PE), early‐secretory phase (ESE), mid‐secretory phase (MSE) and late‐secretory phase (LSE) in published microarray datasets GSE4888 (A) and GSE6364 (B) were statistically analysed and exhibited in the box plots. ns, not significant. *p < 0.05; **p < 0.01.

First, two independent microarray datasets (GSE4888 and GSE6364) downloaded from GEO were utilised to analyse the endogenous expression of chemerin and the cognate receptors in normal human endometrium throughout the menstrual cycle. GSE4888 contains endometrial tissues at the proliferative stage (PE), early‐secretory stage (ESE), mid‐secretory stage (MSE) and late‐secretory stage (LSE) and GSE6364 contains PE, ESE and MSE. Analysis results showed that *RARRES2* levels in ESE and MSE were significantly reduced (*p* < 0.05) when compared with the PE, and *CMKLR1* and *CCRL2* levels in ESE and MSE tended to decrease gradually. There was no significant change of *CMKLR2* in the endometrium at distinct stages (Figure 1A,B). These results preliminarily suggest that the chemerin signalling system changes with the menstrual cycle, and it is relatively inactive in the receptive endometrium.

### 2.2 Molecular Characterisation of Human Endometrial Epithelial 12Z Cells

**FIGURE 2:**
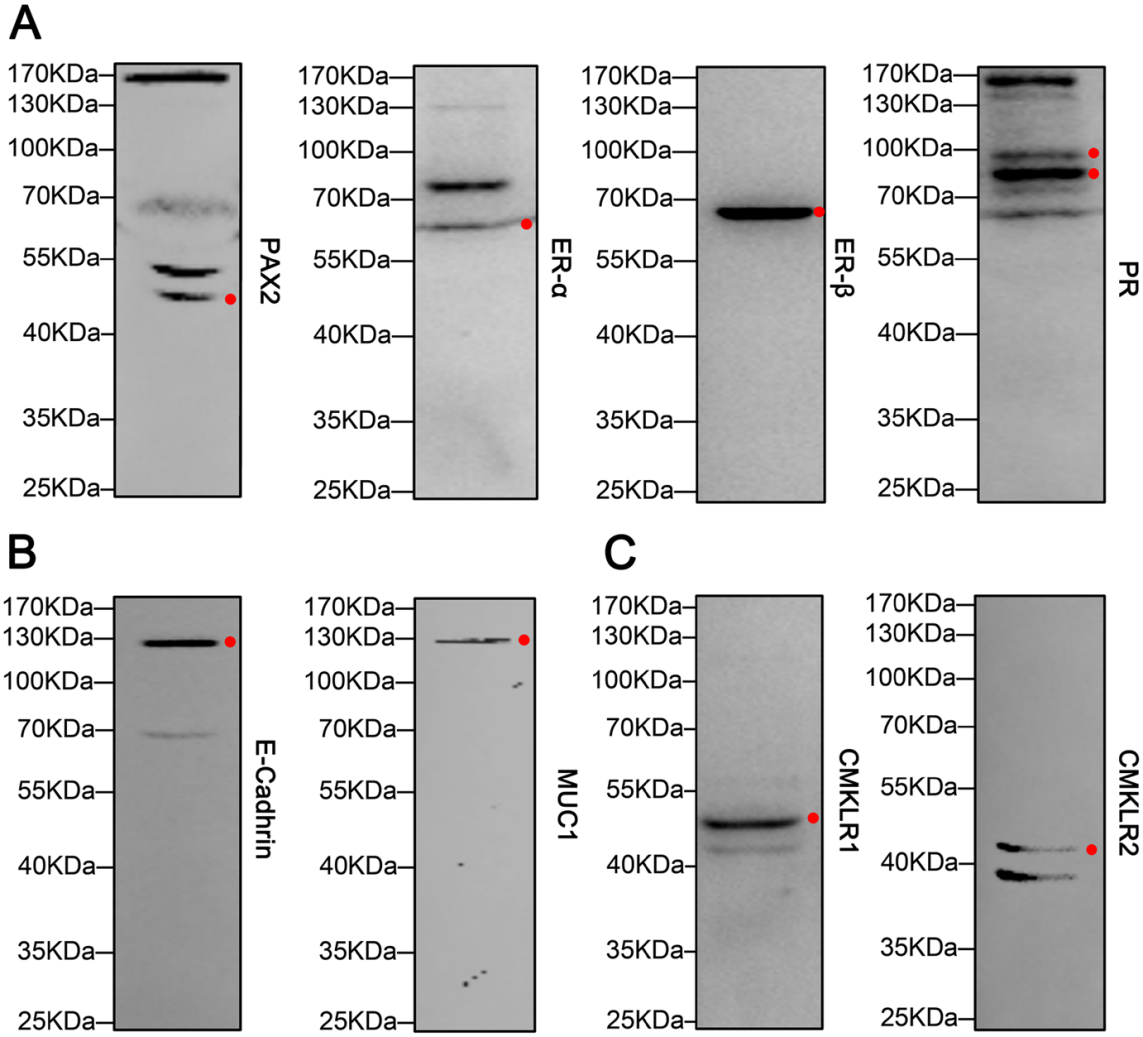
Molecular characterisation of 12Z cells. Immunoblots of endometrial cell markers (PAX2, ERα, Erβ, and PR) (A), epithelial cell markers (E‐cadherin and MUC1) (B), and the functional receptors for chemerin (CMKLR1 and CMKLR2) (C) in 12Z cells. The red dots represent the target protein. The experiments were performed in triplicate, and representative immunoblotting results are shown.

In this study, human endometriotic epithelial 12Z cells were selected as the in vitro model. Before the experiment, we performed Western blot to characterise the cellular origin at the molecular level. Results showed that 12Z cells expressed endometrial cell markers (PAX2, ERα, ERβ and PR) (Figure 2A), epithelial cell markers (E‐cadherin and MUC1) (Figure 2B) indicative of the identity of endometrial epithelial cells (EECs). More importantly, functional receptors (CMKLR1 and CMKLR2) for chemerin were detected (Figure 2C) indicating that 12Z cells can respond to the ligand.

### 2.3 Chemerin Alters the Transcriptome of 12Z Cells

**FIGURE 3:**
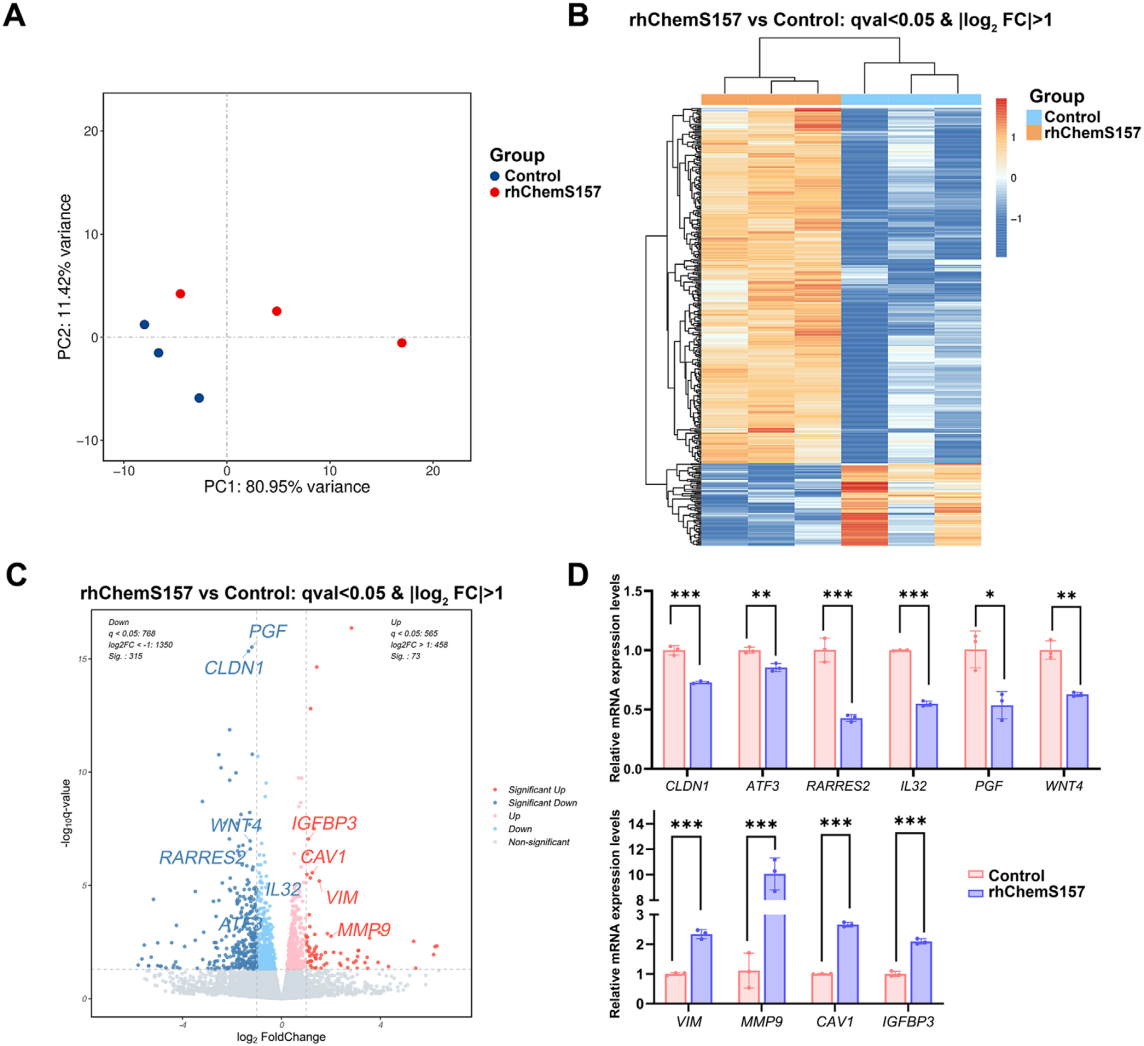
Chemerin alters the transcriptome of 12Z cells. (A) Principal component analysis (PCA) of RNA‐sequencing datasets from 12Z cells treated without (*n* = 3) or with rhChemS157 (*n* = 3). (B) Unsupervised hierarchical clustering of all differentially expressed genes (DEGs) between control and treatment groups. (C) Volcano plot showing the DEGs (red points: Up‐DEGs; blue points: Down‐DEGs). (D) Ten genes (*CLDN1*, *ATF3*, *RARRES2*, *IL32*, *PGF*, *WNT4*, *VIM*, *MMP9*, *CAV1* and *IGFBP3*) highlighted on the volcano plot were further validated by qPCR. All experiments were repeated for three times. *p < 0.05; **p < 0.01; ***p < 0.001.

To explore the effects of chemerin on endometrial cells, we performed RNA‐seq on 12Z cells treated without (Control, *n* = 3) or with recombinant human chemerin protein (Glu21‐Ser157) (rhChem157S, *n* = 3). Principal component analysis (PCA) based on gene expression showed that two groups were separated (Figure 3A), manifesting that rhChem157S alters the global transcriptome level of 12Z cells. By utilising DESeq2 methods, we found 388 significant differentially expressed genes (DEG) (*q* value < 0.05 and |log_2_FoldChange| > 1), with 315 up‐regulated and 73 down‐regulated, in rhChem157S‐treated 12Z cells compared with the controls (Figure 3A,B and Table S1). Among the Top 50 DEGs, we noticed the decreased expression of *RARRES2* indicating a negative feedback regulatory mechanism was exhibited in 12Z cells stimulated by rhChem157S. We also observed the decreased expression of *CLDN1* and increased expression of *VIM* and *MMP9* indicating that rhChem157S promotes the epithelial‐mesenchymal transition (EMT). More importantly, the DEGs contained *ATF3* (Activating transcription factor 3), *PGF* (Placental growth factor), *WNT4* (Wnt Family Member 4), *IL32* (Interleukin 32), *CAV1* (Caveolin‐1) and *IGFBP3* (Insulin‐Like Growth Factor Binding Protein 3), which has been reported to regulate the endometrial receptive functions (Figure 3C). These 10 genes were selected for qPCR validation, and results showed that the expression changes between the two groups were consistent with the transcriptomic analysis results (Figure 3D).

### 2.4 Chemerin Affects Lipid Metabolism and Sialylation of 12Z Cells

**FIGURE 4:**
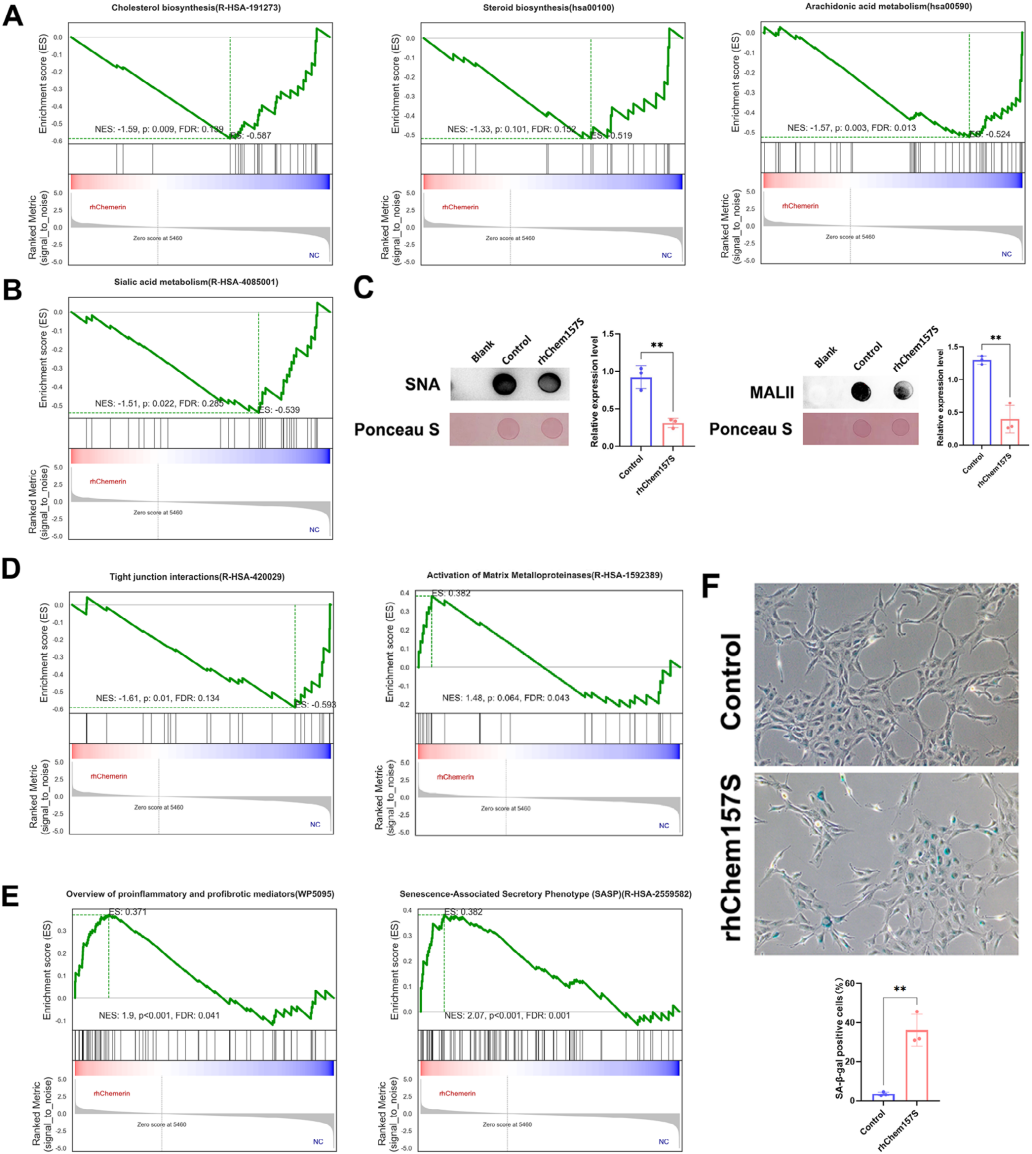
Chemerin influences the biological functions of 12Z cells. (A) GSEA plots showing the enrichment of gene sets of “Cholesterol biosynthesis”, “Steroid hormone biosynthesis” and “Arachidonic acid metabolism”. (B) GSEA plot showing the enrichment of gene sets of “Sialic acid metabolism”. (C) Dot blots of SNA and MAL II in 12Z cells treated with rhChem157S (10 ng/mL) for 48 h. Unprocessed 12Z cells were used as a control group. (D) GSEA plots show the enrichment of gene sets of “Tight junction interactions” and “Activation of Matrix Metalloproteinases”. (E) GSEA plots showing the enrichment of gene sets of “Overview of proinflammatory and profibrotic mediators”, and “Senescence‐Associated Secretory Phenotype”. (F) Senescence‐associated β‐galactosidase (SA‐β‐gal) staining of 12Z cells following a 96‐h incubation with rhChem157S (10 ng/mL). Unprocessed 12Z cells were used as a control group. The experiments were performed in triplicate, and representative results are presented in (C) and (F). Data are shown as mean ± SD. **p < 0.01.

To gain more biological insights from the transcriptomic data, we applied Gene Set Enrichment Analysis (GSEA) based on gene sets documented in the Kyoto Encyclopedia of Genes and Genomes (KEGG), Reactome and Wikipathways. The results of the three databases were considered comprehensively (Table S2), and we found that chemerin markedly affected the glucose, lipid and vitamin metabolism in 12Z cells. Take lipid metabolism as an example, rhChem157S treatment negatively correlates with the terms including “Cholesterol biosynthesis” (*p*: 0.009, FDR: 0.139), “Steroid hormone biosynthesis” (*p*: 0.101, FDR: 0.152) and “Arachidonic acid metabolism” (*p*: 0.003, FDR: 0.013) (Figure 4A). Meanwhile, chemerin regulated the protein and lipid glycosylation in 12Z cells as GSEA results showed that rhChemS157 promoted the “Mucin type O‐glycan biosynthesis” (*p*: 0.046, FDR: 0.142), while inhibiting the “Sialic acid metabolism” (*p*: 0.022, FDR: 0.285) and “Termination of O‐glycan biosynthesis” (*p*: 0.026, FDR: 0.202) (Table S2) (Figure 4B). Glycans, especially the sialic acids terminated glycans, play essential roles in endometrium‐embryo interactions. Therefore, we further validated that rhChemS157 inhibited the α2,3‐sialylation and α2,6‐sialylation levels in 12Z cells by using *Maackia Amurensis* Lectin II (MAL‐II) and *Sambucus Nigra* (SNA), respectively (Figure 4C).

### 2.5 Chemerin Induces the EMT‐Like Process and Cellular Senescence of 12Z Cells

From the cellular biology level, chemerin seems to transform the epithelial characteristics of 12Z cells into a mesenchymal‐like phenotype, as the GSEA results evidenced that rhChem157S treatment is negatively related to “Tight junction interactions” (*p*: 0.01, FDR: 0.134), whereas positively related to “Activation of Matrix Metalloproteinases” (*p*: 0.064, FDR: 0.043) (Figure 4D). Furthermore, GSEA results found rhChem157S stimuli are positively related to “Overview of proinflammatory and profibrotic mediators” (*p* < 0.001, FDR: 0.041), “Senescence‐Associated Secretory Phenotype” (*p* < 0.001, FDR: 0.001), “DNA Damage/Telomere Stress Induced Senescence” (*p* < 0.001, FDR: 0.004), “Oxidative Stress Induced Senescence” (*p* < 0.001, FDR: 0.052) and “Cellular Senescence” (*p* < 0.001, FDR: 0.054) (Table S2) (Figure 4E). Senescence‐associated beta‐galactosidase (SA‐β‐gal) staining assay of 12Z cells further confirmed that rhChem157S promoted the cell aging (Figure 4F), indicating that over‐produced chemerin underpins the inflammaging of uterine endometrium during obesity.

### 2.6 Chemerin Restrains the STAT3 Signalling Pathway in 12Z Cells

**FIGURE 5:**
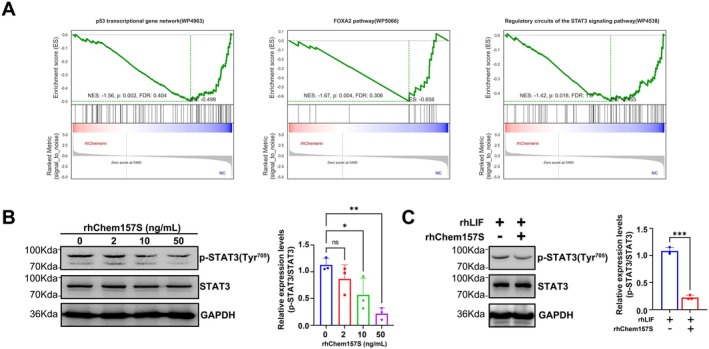
Chemerin restrains the STAT3 signalling pathway in 12Z cells. (A) GSEA plots showing the enrichment of gene sets of “p53 transcriptional gene network”, “FOXA2 pathway”, and “Regulatory circuits of the STAT3 signaling pathway”. (B) Immunoblots of Phospho‐STAT3 (Tyr705), STAT3 and GAPDH in 12Z cells treated with rhChem157S (0, 2, 10 and 50 ng/mL) for 48 h. (C) Immunoblots of Phospho‐STAT3 (Tyr705), STAT3 and GAPDH in 12Z cells pre‐treated without or with rhChem157S (10 ng/mL) for 48 h followed by stimulating with rhLIF (10 ng/mL) for 30 min. GAPDH was used as an internal control. The experiments were performed in triplicate, and representative immunoblotting results are presented. The densitometry of blots was shown in the bar plots. Data are shown as mean ± SD. ns, not significant. *p < 0.05; **p < 0.01; ***p < 0.001.

Through enrichment of the gene sets affiliated to WikiPathways (Table S2), GSEA results showed that rhChem157S stimulation is negatively correlated to “p53 transcriptional gene network” (*p*: 0.002, FDR: 0.404), “FOXA2 pathway” (*p*: 0.004, FDR: 0.306) and “Regulatory circuits of the STAT3 signaling pathway” (*p*: 0.0018, FDR: 1.0) (Figure 5A), which are critical signalling pathways for endometrial receptivity establishment. Although the FDR value is relatively high, we chose to validate the STAT3 signalling pathway because both p53 and FOXA2 were found to be involved in influencing the STAT3 signalling pathway in endometrium [26, 27]. To validate the impact of rhChem157S on STAT3 signalling in 12Z cells, we performed Western blot analysis. Initially, 12Z cells were treated with varying concentrations of rhChem157S for 48 h. Under basal conditions, rhChem157S inhibited the phosphorylation level of STAT3 (p‐STAT3) at Tyr^705^ in a dose‐dependent manner, as shown in Figure 5B. In our subsequent experiments, we employed leukaemia inhibitory factor (LIF), a key inducer of the STAT3 signalling pathway, plays pivotal roles in endometrial receptivity. Notably, pretreatment with rhChem157S weakened rhLIF‐induced p‐STAT3 (Tyr^705^) level in 12Z cells (Figure 5C). These results suggest that chemerin is detrimental for endometrial receptive establishment.

## 3 Discussion

In this study, we analysed the transcriptome profiling of 12Z cells treated with chemerin. The results showed that chemerin significantly affected the expression of several genes closely related to endometrial receptive functions. In addition, GSEA enrichment analysis revealed that chemerin causes a metabolic disturbance, EMT‐like process and cellular senescence, and negatively affects several pathways involved in receptivity establishment.

Among the down‐regulated DEGs, several genes (*ATF3*, *PGF*, *WNT4* and *IL32*) (Figure 3C,D) are closely related to epithelium‐embryo attachment and decidualization. ATF3 is a transcription factor, which has been found to express in human endometrial epithelial and stromal cells. ATF3 promoted the STAT3 signalling pathway through transcriptional regulation of LIF expression and further promoted the receptivity of Ishikawa cells to BeWo‐spheroids in vitro [28]. Importantly, ATF3 is aberrantly low expression in endometria of recurrent implantation failure (RIF) patients. Dysregulation of ATF3 impaired the decidualization and proliferation of stromal cells [29]. PGF is expressed in human endometrial epithelial, decidualising stromal cells and immune cells, and it can be identified in human uterine lavage fluid. rhPGF promotes the development and outgrowth of mouse embryos and enhances the adhesion of human EECs to fibronectin in vitro, indicating an important role of PGF during implantation [30]. WNT4 was localised in the human endometrial epithelium and stromal cells, and its expression in the endometrium of RIF patients is abnormally decreased [31]. Mouse models reveal that WNT4 is required for postnatal uterine development, epithelial differentiation, decidualization and embryo implantation [32]. IL32 regulates the integrin signalling pathway, and rhIL32 promotes the receptivity of AN3CA cells to Jeg‐3 spheroids in vitro [33]. These previous findings imply that ATF3, PGF, WNT4 and IL32 are vital for embryo implantation, whereas chemerin may disrupt uterine receptivity by inhibiting the expression of these genes.

Uterine aging seriously affects the hormone response capacity and receptive function of the endometrium. Oxidative stress, chronic inflammation, fibrosis, DNA damage response, and cellular senescence are considered the core factors that cause uterine aging [34]. GSEA enrichment analysis found that chemerin treatment was positively correlated with “Overview of proinflammatory and profibrotic mediators”, and “Senescence‐Associated Secretory Phenotype” (Figure 4E). SA‐β‐gal staining assay was carried out to ascertain that chemerin promotes the cellular senescence of 12Z cells (Figure 4F). Furthermore, chemerin significantly up‐regulated two genes (*CAV1* and *IGFBP3*) that are reported to participate in senescence in the uterus (Figure 3C,D). Caveolin‐1 is required for the formation of caveolae (invaginations of the plasma membrane) in most cell types and regulates the cellular senescence [35]. Ectopically expression of Caveolin‐1 attenuates the expression of IGFBP1 (decidual cell marker) in decidualizing hESCs, implying that low levels of CAV‐1 should be conducive to decidualization [36]. IGFBP3 is highly released by premature senescent human endometrium‐derived mesenchymal stem cells (hEMSCs) and thus induces senescence of adjacent cells via an autocrine/paracrine pathway [37]. Collectivity, chemerin induces a cellular senescence phenotype of endometrial cells, which may trigger a cascade of dysfunctions.

EMT and its reverse mesenchymal‐to‐epithelial transition (MET) play crucial roles in development, remodels and decidualization of endometrium [38]. During the period of embryo attachment, embryonic factors trigger an EMT‐like process of EECs to allow the subsequent embryo penetration and invasion [39]. In this article, we found that chemerin significantly inhibited the expression of *CLDN1* and up‐regulated the expression of *VIM* and *MMP9*, and the GSEA enrichment assay further showed that chemerin treatment caused EMT‐like process in 12Z cells (Figures 3D and 4D). These results raised three questions: (1) Under normal physiological conditions, whether the embryo expresses and releases chemerin to regulate the EMT of EECs; (2) In obese women of childbearing age, whether overproduced chemerin intervened in the EMT of EECs before the embryo attachment; (3) Overexpression of chemerin was found in some uterine‐related diseases such as endometriosis [40], and whether chemerin affects the lesion tissue invasion through EMT. It is necessary to analyse the function and significance of chemerin in regulating the EMT of EECs based on specific situations in the future.

Proper lipid (fatty acids, cholesterol, etc.) metabolism is required for the maintenance of female reproductive system function, embryonic development and pregnancy [41]. Prostaglandins (PG), the metabolites of arachidonic acid (AA), are well‐known players for successful implantation. Studies showed that deficiency of enzymes involved in PG biosynthesis is related to compromised endometrial receptivity in humans and mice [42]. In this study, GSEA analysis results pointed out that chemerin suppressed the AA metabolism in 12Z cells (Figure 4A). Furthermore, our results also pointed out that chemerin suppressed the cholesterol and steroid hormone biosynthesis in 12Z cells (Figure 4A), which is in line with the previous findings found in granulosa (humans, rats, mice, pigs and bovine) and porcine endometrial cells [25]. Cholesterol is not only a precursor of sex hormones but is also involved in regulating numerous cellular functions [42]. In sum, we found that chemerin induced abnormal lipid metabolism in EECs, which may lead to implantation failure.

Protein glycosylation is a common post‐translational modification involved in intercellular and cell‐extracellular matrix interactions. Glycans at the maternal‐fetal interface, especially the terminal sialic acids (sialylation), play a critical role in the process of embryo implantation [43]. ST3GAL3 (β‐galactoside α2,3‐sialyltransferase 3) is found to be highly expressed in human and mouse endometrial epithelium, and ST3GAL3‐generated α2,3‐sialylation promotes the receptivity in vitro and in vivo [44]. ST6GAL1 (β‐galactoside α2,6‐sialyltransferase 1)‐mediated α2,6‐sialylation has also been evidenced to regulate the uterine lumen closure during implantation in pigs [45]. In this study, GSEA enrichment analysis found that chemerin treatment was negatively correlated with “sialic acid metabolism”, and to the best of our knowledge, we have found for the first time that chemerin simulation inhibited the α2,3‐ and α2,6‐sialylation in 12Z cells (Figure 4B,C). Based on these results, it may be postulated that chemerin negatively affects the epithelium‐embryo attachment by inhibiting the sialylation of EECs.

The GSEA enrichment analysis showed that chemerin inhibits the p53, FOXA2 and STAT3 signalling pathways (Figure 5A), which are documented to play critical roles in endometrial functions in reproduction. Mice with uterine‐specific p53 knockout endow premature uterine senescence and promote preterm birth [46]. Mice with uterine‐specific FOXA2‐deleted are completely infertile [26]. Mice with uterine‐specific STAT3 deficiency exhibited implantation failure phenotype [47]. Given that p53 and FOXA2 also affect the STAT3 pathway through the regulation of LIF [26, 27], therefore, we evaluated the status of STAT3 signalling transduction after chemerin treatment. Immunoblots results showed that rhChem157S suppressed the p‐STAT3 levels at a basal level (Figure 5B), and desensitised the activation of p‐STAT3 in response to rhLIF (Figure 5C). These data further suggest that chemerin is a negative regulator of endometrial receptivity.

Adipokine chemerin, acting as a pivotal regulator of metabolism and inflammation, is involved in influencing reproductive functions, but its specific impact on endometrial function remains unclear [25]. Previous studies have utilised transcriptomic and proteomic methods to study the effects of chemerin on the porcine uterine endometrium during the implantation period. Chemerin was found to influence cell migration, adhesion, immune responses and angiogenesis [48, 49]. The same research group has reported that chemerin affects the expression of angiogenesis‐related factors and enzymes related to hormone synthesis in porcine endometrial cells [50, 51]. In this study, we for the first time applied a human endometrial epithelial cell line as an in vitro model. Additionally, unlike the concentration of chemerin used (400 ng/mL) in previous studies, we chose 10 ng/mL of chemerin to treat the 12Z cells, which is in line with the range of chemerin concentrations found in the plasma of obese patients [23]. It is worth noting that the use of human endometriotic cell line remains suboptimal, we plan to establish endometrial epithelial organoids (EEOs) from human or mouse (wild type or chemerin receptors knockout strains) to further explore the effects of chemerin on endometrial functions. More importantly, the impact of other adipokines on endometrial function warrants further investigation by utilising similar strategy. This could reveal new insights into obesity‐related factors in reproductive health, potentially uncovering new avenues for intervention.

In summary, this study elucidates the effects of adipokine chemerin on endometrial epithelial cells by transcriptomic analysis. Obesity‐derived chemerin may be one of the important causes of endometrial dysfunction. These findings will help expand our understanding of the relationship between obesity and reproduction, and provide potential strategies for intervention treatment.

## 4 Materials and Methods

### 4.1 Cell Culture

12Z cells were obtained from the American Type Culture Collection. 12Z cells were maintained in DMEM/F‐12 (Gibco). All cell conditional medium was supplemented with 10% (v/v) FBS and 1% penicillin–streptomycin. 12Z cells were characterised as mycoplasma negative using the MycoBlue Mycoplasma Detector (Vazyme, Catalogue #: D101‐02) according to the manufacturer's instructions. Cells were cultured in a humidified atmosphere containing 5% CO2 at 37°C. The medium was renewed every 2–3 days.

### 4.2 Real‐Time Quantitative PCR Analysis

Cells were treated with RNAiso Plus reagent (Takara, Catalogue #:9109) for RNA extraction, and the PrimeScriptTM RT Master Mix (Takara, Catalogue #: RR036A) was used to synthesise cDNA. TB Green Premix Ex Taq (Tli RNaseH Plus) (Takara, Catalogue #: RR420L) was used for qPCR. The primers were as follows: *CLDN1*: 5′‐CTT GGC ATG GTG GGG ACT C‐3′ (Forward), 5′‐CTG GCT TGT CGG ATG CAA TTC‐3′ (Reverse); *ATF3*: 5′‐TGC TCA GAG AAG TCG GAA GAA‐3′ (Forward), 5′‐TGG CAC AAA GTT CAT AGG GCA‐3′ (Reverse); *PGF*: 5′‐GAA CGG CTC GTC AGA GGT G‐3′ (Forward), 5′‐ACA GTG CAG ATT CTC ATC GCC‐3′ (Reverse); *WNT4*: 5′‐AGG AGG AGA CGT GCG AGA AA‐3′ (Forward), 5’‐CGA GTC CAT GAC TTC CAG GT‐3′ (Reverse); *IL32*: 5′‐TGG CGG CTT ATT ATG AGG AGC‐3′ (Forward), 5′‐CTC GGC ACC GTA ATC CAT CTC‐3′ (Reverse); *CAV1*: 5’‐GCG ACC CTA AAC ACC TCA AC‐3′ (Forward), 5′‐ATG CCG TCA AAA CTG TGT GTC‐3′ (Reverse); *IGFBP3*: 5′‐AGA GCA CAG ATA CCC AGA ACT‐3′ (Forward), 5′‐GGT GAT TCA GTG TGT CTT CCA TT‐3′ (Reverse); *ACTB*: 5′‐GTT GAG AAC CGT GTA CCA TGT‐3′ (Forward), 5′‐TTC CCA CAA TTT GGC AAG AGC‐3′ (Reverse); *RARRES2*: 5′‐TGG AAG AAA CCC GAG TGC AAA‐3′ (Forward), 5′‐AGA ACT TGG GTC TCT ATG GGG‐3′ (Reverse); *VIM*: 5′‐GCC CTA GAC GAA CTG GGT C‐3′ (Forward), 5′‐GGC TGC AAC TGC CTA ATG AG‐3′ (Reverse); *MMP9*: 5′‐TGT ACC GCT ATG GTT ACA CTC G‐3′ (Forward), 5′‐GGC AGG GAC AGT TGC TTC T‐3′ (Reverse); The reactions were performed using the LightCycler 96 Real‐time PCR System (Roche, Switzerland). Quantified data were normalised to those of *ACTB*, and the relative quantity was calculated using the 2^−ΔΔCT^ method.

### 4.3 Protein Isolation, Immunoblot and Dot Blot Analysis

12Z cells were cultured in 6‐well plates and were either untreated or treated with rhChem157S (R&D System, Catalogue #: 2324‐CM) at different concentrations (2, 10, 50 ng/mL), or with Human Recombinant Leukaemia inhibitory factor (rhLIF) (10 ng/mL) (Peprotech, Catalogue #:300–05). Proteins were extracted by M‐PER Mammalian Protein Extraction Reagent (Thermo Fisher, Catalogue #: 78501), and the concentrations were quantified by Pierce BCA (Thermo Fisher, Catalogue #: 23227). For immunoblot, equal proteins were loaded onto 12% SDS‐PAGE gels and then transferred onto a nitrocellulose (NC) membrane. For dot blot, equal proteins were directly loaded onto an NC membrane. After blocking with 5% non‐fat dry milk at RT for 2 h, the membranes were incubated at 4°C overnight with the primary antibody: anti‐Progesterone receptor (Santa Cruz Biotechnology, Dallas, Texas, USA, Catalogue #: sc‐810, 1/500), anti‐Oestrogen Receptor alpha (Santa Cruz Biotechnology, Catalogue #: sc‐8005, 1/500), anti‐Oestrogen Receptor beta (Invitrogen, Catalogue #: PA1‐311, 1/1000), anti‐ChemR23 (Santa Cruz Biotechnology, Catalogue #: sc‐374,570, 1/500), anti‐GPR1 (Novus Biologicals, Catalogue #: 41082, 1/1000), anti‐MUC1 (Santa Cruz Biotechnology, Catalogue #: sc‐7313, 1/500), anti‐PAX2 (Proteintech, Wuhan, Chain, Catalogue #: 29307‐1‐AP, 1/1000), anti‐E‐cadherin (Proteintech, Catalogue#: 60335‐1‐Ig, 1/1000), anti‐STAT3 (Cell Signalling Technology, Catalogue #: 12640, 1/1000), anti‐Phospho‐STAT3 (Tyr^705^) (Cell Signalling Technology, Catalogue #: 9145, 1/1000), anti‐GAPDH (Proteintech, Catalogue #: 10494–1, 1/2000), Biotinylated Sambucus Nigra (Vector Laboratories, Catalogue #: B‐1305‐2, 1/2000), Biotinylated Maackia Amurensis Lectin II (Vector Laboratories, Catalogue #: B‐1265‐1, 1/2000). Next, the membranes were washed 3 times with TBS‐T followed by incubating with HRP‐conjugated goat anti‐rabbit IgG, HRP‐conjugated goat anti‐mouse IgG, or HRP‐conjugated Streptavidin for 1 h. After washing 3 times with TBS‐T, an enhanced chemiluminescence (ECL) detection system (Bio‐Rad, Hercules, CA, USA) was used to visualise immunoreactive bands. All experiments were repeated three times to ascertain the reliability of the data.

### 4.4 Senescence‐Associated beta‐Galactosidase (SA‐β‐Gal) Staining Assay

Intracellular SA‐β‐gal activity was visualised using the Senescence β‐Galactosidase Staining Kit (Beyotime, Catalogue #: C0602) following the provided protocol. Briefly, cells were washed with PBS after the aspiration of the culture medium, fixed with β‐galactosidase staining fixative at RT for 15 min, and then washed again three times with PBS. Subsequently, the staining working solution was added, and the cells were incubated overnight at 37°C. Stained cells were recorded under an optical microscope. SA‐β‐gal stainigng assay were performed three independent biological replicates. For statistical analysis, we randomly selecting 3–5 fields per group in each experiment to count the positive cells.

### 4.5 RNA Isolation and Library Preparation

12Z cells were cultured in 6‐well plates, and treated with 10 ng/mL rhChem157S (R&D System, Catalogue #: 2324‐CM) for 48 h. Total RNA was extracted using the TRIzol reagent (Invitrogen, CA, USA, Catalogue #: 15596026CN) according to the manufacturer's protocol. RNA purity and quantification were evaluated using the NanoDrop 2000 spectrophotometer (Thermo Scientific, USA). RNA integrity was assessed using the Agilent 2100 Bioanalyzer (Agilent Technologies, Santa Clara, CA, USA). Then the libraries were constructed using VAHTS Universal V6 RNA‐seq Library Prep Kit according to the manufacturer's instructions. The transcriptome sequencing and analysis were conducted by OE Biotech Co. Ltd. (Shanghai, China).

### 4.6 RNA Sequencing and Differentially Expressed Genes Analysis

The libraries were sequenced on an Illumina Novaseq 6000 platform and 150 bp paired‐end reads were generated. About 55.96 M raw reads for each sample were generated. Raw reads of fastq format were firstly processed using fastp [52] and the low‐quality reads were removed to obtain the clean reads. Then about 47.13 M clean reads for each sample were retained for subsequent analyses. The clean reads were mapped to the reference genome (NCBI_GRCh38.p13) using HISAT2 [53]. FPKM [54] of each gene was calculated and the read counts of each gene were obtained by HTSeq‐count [55]. PCA analysis was performed using R (v 3.2.0) to evaluate the biological duplication of samples.

Differential expression analysis was performed using the DESeq2 [56]. *q* value < 0.05 and foldchange > 2 was set as the threshold for significantly differential expression genes (DEGs). Hierarchical cluster analysis of DEGs was performed using R (v 3.2.0) to demonstrate the expression pattern of genes in different groups and samples. The heat map and volcano map were drawn to show the expression of up‐regulated or down‐regulated DEGs using R (v 3.2.0).

Gene Set Enrichment Analysis (GSEA) was performed using GSEA software [57, 58]. The analysis used predefined gene sets affiliated with KEGG, Reactome and WikiPathways, and the genes were ranked according to the degree of differential expression in the two types of samples. Then it is tested whether the predefined gene set was enriched at the top or bottom of the ranking list.

### 4.7 Bioinformatics Approach

The GSE4888 and GSE6364 datasets were downloaded from the GEO DataSets database (www.ncbi.nlm.nih.gov/gds). GSE4888 and GSE6364 contained the microarray data of endometrial biopsies from women with normal endometrial pathology and benign gynaecological diseases from an Affymetrix GPL570 platform. In this article, we excluded six endometrial samples with ambiguous histology in GSE4888 and selected 16 normal endometrial samples in GSE6364. The “Affy” package in R software (version 4.2.0; https://www.r‐project.org/) was used to correct the background and normalise the raw data, and the expression values were then obtained.

### 4.8 Statistical Analysis

Each experiment (qPCR, Western blot, Dot blot and SA‐β‐gal staining assay) was conducted with three biological replicates. For the Western blot assay, we conducted three biological replicates and calculated the grayscale values and band intensities for both groups using ImageJ software. Data are presented as mean ± SD. All statistical analyses were performed using GraphPad Prism 9.0 (La Jolla, CA, USA). Data normality and homogeneity of variances were assessed using the Shapiro–Wilk test and Bartlett's test, respectively. Statistical analysis between two groups was performed using two‐tailed unpaired Student's *t*‐test, and analysis between multiple groups was conducted by one‐way analysis of variance (ANOVA). *p < 0.05; **p < 0.01; ***p < 0.001.

## Author Contributions

**Ming Yu:** Conceptualization, Methodology, Software, Validation, Investigation, Resources, Data Curation, Writing – Original Draft Preparation, Writing – Review and Editing, Visualization, Supervision, Project Administration and Funding Acquisition. **Yichun Wang:** Methodology. **Jinxuan Cai:** Software. **Xinyue Dong:** Validation. **Wang Hao:** Formal Analysis. **Zichen Sun:** Formal Analysis. **Tianxia Xiao:** Investigation. **Jie Chen:** Resources. **Mengxia Li:** Data Curation. **Chunhua Shan:** Writing – Review and Editing. **Yang Dong:** Writing – Review and Editing. **Jian V. Zhang:** Supervision, Project Administration and Funding Acquisition.

## Conflicts of Interest

The authors declare no conflicts of interest.

## Supporting information


Table S1.

Table S2.

## Data Availability

The original contributions presented in the study are included in the article/Supporting Information. The datasets supporting the conclusions of this article are available in GEO: GSE254152 (https://www.ncbi.nlm.nih.gov/geo/query/acc.cgi?acc=GSE254152).
